# Augmentation of leaf color parameters, pigments, vitamins, phenolic acids, flavonoids and antioxidant activity in selected *Amaranthus tricolor* under *s*alinity stress

**DOI:** 10.1038/s41598-018-30897-6

**Published:** 2018-08-17

**Authors:** Umakanta Sarker, Shinya Oba

**Affiliations:** 1 The United Graduate School of Agricultural Science, Laboratory of Field Science, Faculty of Applied Biological Sciences, Gifu University, Yanagido 1-1, Gifu, Japan; 2 Department of Genetics and Plant Breeding, Faculty of Agriculture, Bangabandhu Sheikh Mujibur Rahman Agricultural University, Gazipur, 1706 Bangladesh

## Abstract

*Amaranthus tricolor* genotype VA13 was evaluated under four salinity stress in terms of color parameters, leaf pigments, β-carotene, vitamin C, TPC, TFC, TAC, phenolic acids and flavonoids. Salinity stress significantly increases all the studied traits. The increments of all these compounds were high under moderate and severe salinity stress compared to control condition. In this study, *trans*-cinnamic acid was newly identified phenoic acid in *A. tricolor*. Salicylic acid, vanilic acid, *trans*-cinnamic acid, gallic acid, chlorogenic acid, rutin, isoquercetin and *m*-coumaric acid were the most abundant phenolic compounds of amaranth that increased with the severity of salinity stress. *A. tricolor* leaves are good source of pigments, β-carotene, vitamin C, bioactive compounds, phenolic acids, flavonoids and antioxidants. In salt-stressed amaranth, correlation studies revealed strong antioxidant activity of leaf pigments, β-carotene, vitamin C, TPC, TFC. These bioactive compounds played a vital role in scavenging ROS and could be beneficial to human nutrition by serving as a good antioxidant and antiaging source in human health benefit. *A. tricolor* cultivated under salinity stress conditions can contribute a high quality of the final product in terms of leaf pigments, bioactive compounds, phenolic acids, flavonoids and antioxidants. It can be a promising alternative crop in saline-prone areas.

## Introduction

Salinity, one of the major abiotic stress and serious threat to global food security. It prohibits the cultivation of vegetables in many areas in the globe. It affects plants by creating nutritional imbalance, osmotic stress, water deficiency, and oxidative stress^1^. Moreover, previous studies demonstrated that high salinity changes the level of secondary metabolites in plants, including pigments, phenolic compounds and flavonoids, enhanced plant defense mechanisms against oxidative stress^2^. Salinity aggravates overproduction of reactive oxygen species (ROS) that results in oxidative damage by oxidizing proteins, lipids and DNA and other cellular macromolecules^3^. Plants have an excellent non-enzymatic network of ROS detoxification system through AsA, β-carotene and carotenoids, phenolic compounds and flavonoids^3^.

*Amaranthus tricolor* is an excellent source of leaf pigments, β-carotene, vitamin C, phenolic acids, flavonoids and antioxidant capacity that had a great importance for the food industry as most of them are natural antioxidants and detoxify ROS in the human body^4,5^. Hence, salt-stressed plants could economically be potential sources of antioxidants in the human life. These natural antioxidants play an important role in the human diet as involve in defense against several diseases like cancer, atherosclerosis, arthritis, cataracts, emphysema, and retinopathy, neuro-degenerative and cardiovascular diseases^5–8^. *A. tricolor* is a well-adapted leafy vegetable to different biotic and abiotic stresses and has multipurpose uses. Different factors such as biological, environmental, biochemical, physiological, ecological, and evolutionary processes are involved in the quantitative and qualitative improvement of natural antioxidants of this species of which, salinity stress can rapidly boost up the content of natural antioxidants^9^. There are few reports related to the effect of salinity stress on leaf pigments, vitamins, phenolic acids, flavonoids and antioxidant capacity in different crops including leafy vegetables.

Salt stress elevates vitamin C, phenolics, flavonoids and antioxidant activity in *Cichorium spinosum*^10^. Alam *et al*.^11^ observed different levels of salinity treatment resulted in 8–35% increase in TPC; about 35% increase in TFC; and 18–35% increase in FRAP activity in purslane. Lim *et al*.^12^ reported that buckwheat treated with 10, 50, 100, and 200 mM NaCl concentrations result in an increase of phenolic compounds and carotenoids in the sprouts compared to the control (0 mM). The buckwheat sprouts treated with 10, 50, and 100 mM NaCl after 7 d of cultivation were 57%, 121%, and 153%, higher phenolic content than that of the control condition, respectively. In plants, polyphenol synthesis and accumulation are mostly stimulated in response to salinity^13^. Thus, salt-stressed plants might represent potential sources of polyphenols. To our knowledge, there is no information on *A. tricolor* in response to salinity stress in terms of leaf pigments, β-carotene, vitamin C, phenolic acids, flavonoids and antioxidant capacity. In our previous studies, we selected some antioxidant enriched and high yield potential genotypes^14–21^. Therefore, in present investigation, high antioxidant enriched and high yield potential genotype VA13 were evaluated to study the response of leaf pigments, β-carotene, vitamin C, phenolic acids, flavonoids and antioxidant capacity under four salinity stress.

## Results and Discussion

### Effect of salinity on leaf color parameters and leaf pigments

**Figure 1:**
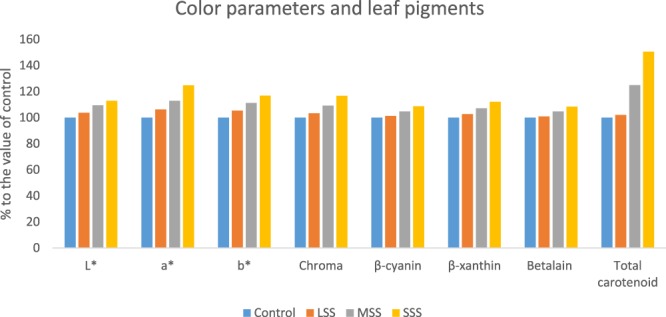
Comparison of color parameters and leaf pigments (% to the value of control) under four salinity levels: Control (No saline water), LSS (Low salinity stress), MSS (Moderate salinity stress) and SSS (Severe salinity stress) in selected *A. tricolor* genotype; L*, Lightness; a*, Redness/greenness; b*, Yellowness/blueness.

Leaf color parameters and leaf pigments under different salinity stress are presented in Table 1. Leaf color is one of the most important parameters for consumers, playing a crucial role in choice making, preference and acceptability of the product, and may also be considered as an indicator for estimating the antioxidant properties of the leafy vegetables^22^. High redness and yellowness values recorded in the genotype VA13 could be expected since it is characterized by the presence of the high pigments (anthocyanins, carotenoids, β-cyanin, β-xanthin and betalain). The results obtained in the present study were fully agreed with the results of Colonna *et al*.^22^. L*, a*, b*, chroma, β-cyanin, β-xanthin, betalain, and total carotenoids were remarkably increased with the severity of salinity stress in the order, Control (No saline water) < Low salinity stress (LSS) < Moderate salinity stress (MSS) < Severe salinity stress (SSS). At LSS, MSS and SSS conditions, L*, a*, b*, chroma, β-cyanin, β-xanthin, betalain and total carotenoids were increased by (4%, 6%, 5%, 3%, 1% 2%, 0.91% & 2%), (10%, 13%, 11%, 9%, 5% 7%, 5% & 24%) and (13%, 25%, 17%, 17%, 9% 12%, 8% & 50%), respectively compared to control condition (Fig. 1). Lim *et al*.^12^ observed continuous increment in the level of carotenoids in response to all NaCl concentrations tested. They reported the greatest difference between the carotenoid content with 50 or 100 mM NaCl which was higher double than that of control sprouts, while treatment with 10 or 200 mM NaCl resulted 40% increase in carotenoids. Unlike other biotic and abiotic stresses, salinity stress induces biosynthesis of abscisic acid (ABA) from carotenoids via mevalonic acid pathway in order to regulate plant development in response to salinity tolerance. Thus, due to NaCl treatment, accumulation of carotenoids in the sprouts might be due to stimulation of the mevalonic acid pathway^12^. Alam *et al*.^11^ reported both increment and decrement in total carotenoid contents in different accessions of purslane with the severity of salinity stress.

**Table 1:** Effect of salinity on leaf color parameters and leaf pigments in selected *A. tricolor* genotype.

Salinity stress	Color parameters				Antioxidant leaf pigments			
	L*	a*	b*	Chroma	β-cyanin (ng g^−1^)	β-xanthin (ng g^−1^)	Betalain (ng g^−1^)	Total carotenoids (mg 100 g^−1^)
Control (No saline water)	31.16 ± 1.85a	10.12 ± 0.87a	3.56 ± 0.28a	12.46 ± 0.52a	624.75 ± 2.54a	266.44 ± 2.81a	902.62 ± 4.52a	35.75 ± 1.24a
Low salinity stress (LSS)	32.34 ± 1.92b	10.76 ± 0.99b	3.75 ± 0.32b	12.88 ± 0.67b	632.83 ± 3.08b	273.72 ± 3.24b	910.87 ± 4.22b	36.52 ± 1.35b
Moderate salinity stress (MSS)	34.12 ± 2.05c	11.42 ± 1.12c	3.96 ± 0.24c	13.62 ± 0.46c	654.62 ± 3.28c	285.68 ± 4.02c	945.56 ± 3.57c	44.68 ± 1.57c
Severe salinity stress (SSS)	35.16 ± 2.14d	12.63 ± 1.02d	4.16 ± 0.22d	14.54 ± 0.44d	678.92 ± 2.98d	298.84 ± 3.87d	978.42 ± 3.92d	53.87 ± 0.98d

Different letters in a column are differed significantly by Duncan Multiple Range Test (P < 0.01).

### Impact of salinity on β-carotene, vitamin C, TPC, TFC and TAC

**Figure 2:**
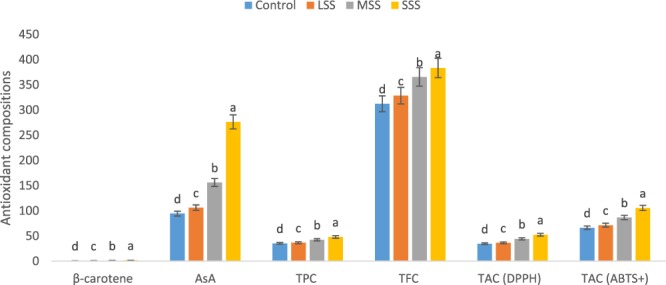
Response to β-carotene, vitamin C, TPC, TFC and TAC under four salinity levels: Control (No saline water), LSS (Low salinity stress), MSS (Moderate salinity stress), SSS (Severe salinity stress) in selected *A. tricolor* genotype; β-carotene (mg g^−1^), AsA, vitamin C (mg 100 g^−1^); TPC, Total polyphenol content (GAE µg g^−1^ dw); TFC, Total flavonoid content (RE µg g^−1^ dw); TAC (DPPH), Total antioxidant capacity (DPPH) (TEAC µg g^−1^ dw); TAC (ABTS^+^), Total antioxidant capacity (ABTS^+^) (TEAC µg g^−1^ dw); (n = 6), different letters are differed significantly by Duncan Multiple Range Test (P < 0.01).

**Figure 3:**
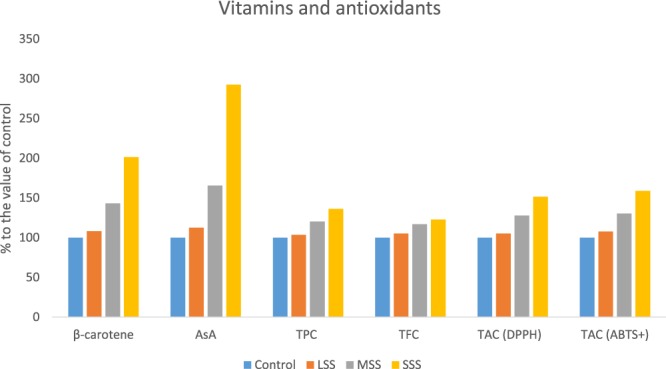
Response to vitamins, TPC, TFC and TAC, (% to the value of control) under four salinity levels: Control (No saline water), LSS (Low salinity stress), MSS (Moderate salinity stress) and SSS (Severe salinity stress) in selected *A. tricolor* genotype; β-carotene (mg g^−1^), AsA, Vitamin C (mg 100 g^−1^); TPC, Total polyphenol content (GAE µg g^−1^ dw); TFC. Total flavonoid content (RE µg g^−1^ dw); TAC (DPPH), Total antioxidant capacity (DPPH) (TEAC µg g^−1^ dw); TAC (ABTS^+^), Total antioxidant capacity (ABTS^+^) (TEAC µg g^−1^ dw).

β-carotene, vitamin C, total polyphenol content (TPC), total flavonoid content (TFC) and total antioxidant capacity (TAC) of the genotype of *A. tricolor* were significantly affected by salinity levels (Fig. 2). The significant increase in β-carotene, vitamin C, TPC, TFC, TAC (DPPH) and TAC (ABTS^+^) due to salinity stress were found in the order: Control < LSS < MSS < SSS. At LSS, MSS and SWS conditions, β-carotene, vitamin C, TPC, TFC, TAC (DPPH) and TAC (ABTS^+^) were increased by (8%, 13%, 4%, 5%, 5% and 8%), (43%, 66%, 20%, 17%, 28% and 30%) and (101%, 192%, 36%, 23%, 52% and 59%), compared to control condition, respectively (Fig. 3). β-carotene, vitamin C, TPC, TFC, TAC (DPPH) and TAC (ABTS^+^) had the highest values under SSS condition, while β-carotene, vitamin C, TPC, TFC, TAC (DPPH) and TAC (ABTS^+^) were observed the lowest in control condition. Petropoulos *et al*.^10^ found the elevated response of phenolics, flavonoids and antioxidant activity with the increase in salt stress in *Cichorium spinosum*. Alam *et al*.^11^ reported that different levels of salinity treatment resulted 8–35% increases in TPC; about 35% increase in TFC; and 18–35% increases in FRAP activity in purslane. Lim *et al*.^12^ reported that buckwheat treated with 10, 50, and 100 mM after 7 d of cultivation were 57%, 121%, and 153%, higher phenolic content than that of the control, respectively. Ahmed *et al*.^23^ reported increment in phenolics and TAC (FRAP) with increasing NaCl concentrations in barley. In contrast, Neffati *et al*.^24^ found decrement in polyphenols and TAC (DPPH) with increasing NaCl concentrations in coriander.

### Influence of salinity on phenolic acids and flavonoids

Data on retention time, λmax, molecular ion, main fragment ions in MS^2^ and tentative compound identification for phenolic compounds are presented in Table 2. The values of phenolic acids and flavonoids components separated though LC from the genotype VA13 was compared with ion masses of standard phenolic acids and flavonoids by observing the particular peaks of the corresponding components. Totally, sixteen phenolic compounds were identified including six hydroxybenzoic acids, seven hydroxycinnamic acids and three flavonoids. In this study, *trans*-cinnamic acid was newly identified phenolic acid in *A. tricolor*. Except for *trans*-cinnamic acid, Khanam and Oba^25^ in red and green amaranths, Khanam *et al*.^26^ in eight different leafy vegetables including amaranths described the rest 15 phenolic acids and flavonoids with normal cultivation practices. However, an attempt was made for the first time to evaluate the effect of sixteen phenolic acids and flavonoids of *A. tricolor* under four salinity stress. Quantification of identified phenolic compounds in selected *Amaranthus tricolor* leaves under four salinity stress are presented in Table 3. Considering phenolic acids and flavonoids, hydroxybenzoic acids having one functional carboxylic acid were the most plentiful compounds in this genotype. Within hydroxybenzoic acids, salicylic acid was found to be as one of the main phenolic acids followed by vanilic acid and gallic acid. Gallic acid and *p*-hydroxybenzoic acid content of the genotype VA13 under control condition were higher than *A. tricolor* genotypes of Khanam *et al*.^26^. Regarding hydroxycinnamic acids, chlorogenic acid was the most abundant compound followed by *trans*-cinnamic acid and *m*-coumaric acid. A good amount of caffeic acid, *p*-coumaric acid, ferulic acid were also observed in this genotype. The genotype VA13 had higher caffeic acid and *m*-coumaric acid under control condition compared to *A. tricolor* genotypes of Khanam *et al*.^26^. The hydroxycinnamic acids synthesized from phenylalanine are the most extensively disseminated phenolic acids in plant tissues^27^. In plants, flavonoids occasionally occur as a glycone, although the most common forms are glycoside derivatives. These compounds account for 60% of total dietary phenolic compounds^28,29^. Flavonols are the most prevalent flavonoids in the plant kingdom and glycosides of quercetin are the most predominant naturally occurring flavonols^28^. In this investigation, the flavonoids, rutin (quercetin-3-rutinoside) and isoquercetin (quercetin-3-glucoside) were the most abundant in this genotype. The genotype VA13 exhibited higher rutin (quercetin-3-rutinoside) content under control condition in comparison to *A. tricolor* genotypes of Khanam *et al*.^26^.

**Table 2:** Retention time (Rt), wavelengths of maximum absorption in the visible region (λ_max_), mass spectral data and tentative identification of phenolic compounds in selected *Amaranthus tricolor* leaves.

Peak no	Rt (min)	λ_max_ (nm)	Molecular ion [M − H]^−^ (m/z)	MS^2^ (m/z)	Identity of tentative compounds
1	9.1	254	169	169.2	3,4-5 Trihydroxybenzoic acid
2	30.6	254	167	167.2	4-hydroxy-3-methoxybenzoic acid
3	34.8	254	197	197.1	4-Hydroxy-3,5-dimethoxybenzoic acid
4	31.5	254	137	137.2	4-hydroxybenzoic acid
5	48.2	254	137	137.2	2-Hydroxybenzoic acid
6	52.5	254	301	301.1	(2,3,7,8-tetrahydroxy-chromeno [5,4,3-cde]chromene-5,10-dione
7	32.0	280	179	179.1	3,4-Dihydroxy-trans-cinnamate
8	31.1	280	353	353.2	3-(3,4-Dihydroxycinnamoyl) quinic acid
9	42.0	280	163	163.1	4-hydroxycinnamic acid
10	47.9	280	193	193.2	4-hydroxy-3-methoxycinnamic acid
11	49.6	280	163	163.3	3-hydroxycinnamic acid
12	49.0	280	223	223.2	4-Hydroxy-3,5-dimethoxycinnamic acid
13	67.3	280	147	147.1	3-Phenylacrylic acid
14	54.3	360	463	463.3	Quercetin-3-glucoside
15	53.3	360	463	463.5	Quercetin-3-galactoside
16	53.0	360	609	609.4	Quercetin-3-rutinoside

**Table 3:** Quantification of identified phenolic compounds (µg g^−1^ FW) in selected *Amaranthus tricolor* leaves under four salinity stress.

Phenolic group	Compound	Control (No NaCl)	LSS (25 mM NaCl)	MSS (50 mM NaCl)	SSS (100 mM NaCl)
**Hydroxybenzoic acid**					
Gallic acid	3,4–5 Trihydroxybenzoic acid	6.64 ± 0.05c	6.67 ± 0.06c	8.46 ± 0.06b	9.39 ± 0.08a
Vanilic acid	4-hydroxy-3-methoxybenzoic acid	9.40 ± 0.12c	9.37 ± 0.09c	12.65 ± 0.08b	14.89 ± 0.22a
Syringic acid	4-Hydroxy-3,5-dimethoxybenzoic acid	1.46 ± 0.01b	1.26 ± 0.02d	1.43 ± 0.01c	1.52 ± 0.02a
*p*-hydroxybenzoic acid	4-hydroxybenzoic acid	2.75 ± 0.02c	2.76 ± 0.03c	3.87 ± 0.02b	4.24 ± 0.01a
Salicylic acid	2-Hydroxybenzoic acid	16.53 ± 0.42d	17.85 ± 0.24c	24.87 ± 0.35b	28.61 ± 0.61a
Ellagic acid	(2,3,7,8-tetrahydroxy-chromeno [5,4,3-cde]chromene-5,10-dione	1.16 ± 0.03c	1.23 ± 0.05b	2.36 ± 0.06a	2.38 ± 0.03a
**Total benzoic acids**		37.95	39.14	53.63	61.03
**Hydroxycinnamic acid**					
Caffeic acid	3,4-Dihydroxy-trans-cinnamate	1.46 ± 0.03c	1.45 ± 0.02c	1.83 ± 0.04b	2.58 ± 0.06a
Chlorogenic acid	3-(3,4-Dihydroxycinnamoyl) quinic acid	7.38 ± 0.32d	7.98 ± 0.52c	9.82 ± 0.28b	12.65 ± 0.48a
*p*-coumaric acid	4-hydroxycinnamic acid	1.16 ± 0.01d	1.25 ± 0.01c	2.53 ± 0.02b	2.62 ± 0.03a
Ferulic acid	4-hydroxy-3-methoxycinnamic acid	1.20 ± 0.02c	1.16 ± 0.02c	2.05 ± 0.04b	3.19 ± 0.05a
*m*-coumaric acid	3-hydroxycinnamic acid	2.87 ± 0.05c	2.87 ± 0.06c	5.25 ± 0.04b	7.36 ± 0.03a
Sinapic acid	4-Hydroxy-3,5-dimethoxycinnamic acid	0.35 ± 0.01b	0.36 ± 0.01b	0.43 ± 0.01a	0.45 ± 0.01a
*Trans*-cinnamic acid	3-Phenylacrylic acid	6.85 ± 0.02b	6.86 ± 0.01b	6.89 ± 0.02a	6.92 ± 0.03a
**Total cinnamic acids**		21.28	21.93	28.80	35.77
**Flavonoids**					
Iso-quercetin	Quercetin-3-glucoside	4.66 ± 0.21c	4.80 ± 0.24c	7.23 ± 0.16b	9.24 ± 0.18a
Hyperoside	Quercetin-3-galactoside	1.35 ± 0.02b	1.33 ± 0.01b	2.43 ± 0.01a	2.44 ± 0.02a
Rutin	Quercetin-3-rutinoside	6.62 ± 0.11d	6.74 ± 0.09c	8.87 ± 0.08b	9.92 ± 0.14a
**Total flavonoids**		**12.63**	**12.87**	**18.53**	**21.60**
**Total phenolic acids**		**59.23**	**61.07**	**81.43**	**96.80**
**Total phenolic index**		**71.86**	**73.94**	**100.96**	**118.40**

Different letters in a row are differed significantly by Duncan Multiple Range Test (P < 0.01); (n = 6).

**Figure 4:**
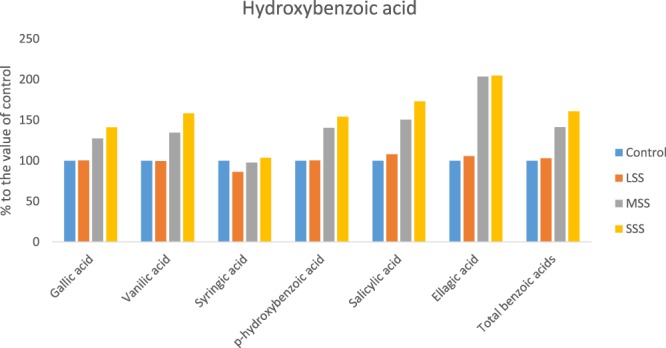
Changes of hydroxybenzoic acid compositions (µg g^−1^ FW) (% to the value of control) under four salinity levels: Control (No saline water), LSS (Low salinity stress), MSS (Moderate salinity stress) and SSS (Severe salinity stress) in selected *A. tricolor* genotype.

**Figure 5:**
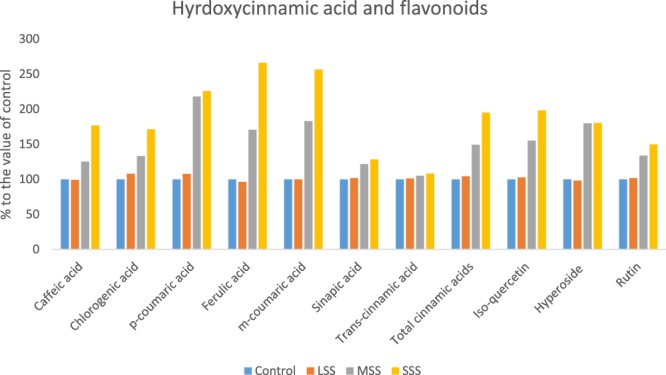
Changes of hydroxycinnamic acid and flavonoid compositions (µg g^−1^ FW) (% to the value of control) under four salinity levels: Control (No saline water), LSS (Low salinity stress), MSS (Moderate salinity stress) and SSS (Severe salinity stress) in selected *A. tricolor* genotype.

Three hydroxybenzoic acids (Gallic acid, vanilic acid and *p*-hydroxybenzoic acid); three hydroxycinnamic acid (Caffeic acid, ferulic acid and *m*-coumaric acid) and flavonoids iso-quercetin had no significant differences in their composition between control and LSS, however, the compositions of these acids were increased significantly from MSS to SSS. In MSS and SSS, the composition of these phenolic acids and flavonoids were increased by (27%, 35%, 41%, 25%, 71% 83% and 55%) and (41%, 58%, 54%, 77%, 166% 156% and 98%); respectively (Figs 4 and 5). Salicylic acid, chlorogenic acid, *p-*coumaric acid and rutin were remarkably increased with the severity of salinity stress (Control < LSS < MSS < SSS). In LSS, MSS and SSS, the concentration of these phenolic acids and flavonoids were increased by (8%, 8%, 8% and 2%); (50%, 33%, 18% and 34%) and (73%, 71%, 26% and 50%); respectively (Figs 4 and 5). Sinapic acid, *trans-*cinnamic acid, and hyperoside had no significant differences in their composition at control and LSS condition, however, the compositions of these acids were increased significantly under MSS or SSS condition compared to control and LSS condition. The composition of these acids under MSS or SSS was statistically similar. The ellagic acid content was significantly increased in the order: Control < LSS < MSS = SSS by 6% and 103% at LSS and MSS or SSS, respectively (Figs 4 and 5); while syringic acid concentration was increased in the order: LSS < MSS < Control < SSS. Except for syringic acid, all the phenolic acids and flavonoids exhibited low concentrations under control condition, whereas these acids had the highest concentrations under SSS condition. Lim *et al*.^12^ reported that buckwheat sprouts treated with 10, 50, and 100 mM NaCl after 7 d of cultivation were 57%, 121%, and 153%, higher phenolic content than that of the control condition, respectively. The total phenolic compounds ranged from 65.86 to 112.40 µg g^−1^ extract, with a significant and sharp increment from control to SSS in the following order: Control < LSS < MSS < SSS. Klados and Tzortzakis^30^ reported a significant increase in total phenolic acids and flavonoids content with increasing salinity in *Cichorium spinosum*. Similarly, total phenolic acids and total flavonoids ranged from 53.23 to 90.80 and 12.63 to 21.60 µg g^−1^ extract, respectively with significantly and sharply increased from control to SSS (Control < LSS < MSS < SSS). Petropoulos *et al*.^10^ found elevated response of phenolic acids and flavonoids with the increase in salt stress in *Cichorium spinosum*. Ahmed *et al*.^23^ reported increment of phenolic acids with increasing NaCl concentrations in barley.

### Correlation studies

The correlation coefficient among β-cyanin, β-xanthin, betalain, total carotenoids, β-carotene, ascorbic acid, TPC, TAC (DPPH) and TAC (ABTS^+^) are presented in Table 4. β-cyanin, β-xanthin and betalain had highly significant positive correlations among each other and with TPC, TAC (DPPH) and TAC (ABTS^+^). Significant association between TAC (DPPH) and TAC (ABTS^+^) represented a crucial role of β-cyanin, β-xanthin and betalain in the total antioxidant activity of *A. tricolor* leaves. Total carotenoids displayed significant relationships with β-carotene, vitamin C, TFC, TAC (DPPH) and TAC (ABTS^+^) demonstrating the vital role of carotenoid pigments in the antioxidant activity. β-carotene showed highly significant interrelationships with vitamin C, TAC (DPPH) and TAC (ABTS^+^) and significant association with TPC and TFC. It indicated that increase in β-carotene was directly related to the increment of TPC, TFC, TAC (DPPH) and TAC (ABTS^+^). Similarly, vitamin C revealed significant interrelationship with TAC (DPPH) and TAC (ABTS^+^). Both β-carotene and vitamin C played a vital role in the antioxidant activity of *A. tricolor*. In contrast, vitamin C exerted negligible insignificant association with TPC and TFC. Jimenez-Aguilar and Grusak^31^ found similar results for vitamin C in different species of *Amaranthus*. TPC, TFC and TAC (DPPH) were found significantly interrelated among each other. Alam *et al*.^11^ also reported significant correlation of carotenoids, TPC, TFC with TAC (FRAP) in salt-stressed purslane. Significant positive interrelationship of TPC, TFC, TAC (DPPH) and TAC (ABTS^+^) signify that TPC, TFC had strong antioxidant activity. Similarly, significant positive association between TAC (DPPH) and TAC (ABTS^+^) confirmed the validation of antioxidant capacity of *A. tricolor* by two different methods of antioxidant capacity measurement. Leaf pigments, β-carotene, vitamin C, TPC and TFC had strong antioxidant activity as these bioactive compounds showed significant association with TAC (DPPH) and TAC (ABTS^+^).

**Table 4:** Correlation coefficient for antioxidant leaf pigments, vitamins, TPC, TFC and TAC in selected *A. tricolor* genotype.

	β-xanthin (ng g^−1^)	Betalain (ng g^−1^)	Total carotenoids (mg 100 g^−1^)	β-carotene (mg g^−1^)	Vitamin C (mg 100 g^−1^)	TPC (GAE µg g^−1^ dw	TFC (RE µg g^−1^ dw)	TAC (DPPH) (TEAC µg g^−1^ dw)	TAC (ABTS^+^) (TEAC µg g^−1^ dw)
β-cyanin	0.96**	0.95**	0.32	0.37	0.18	0.87**	−0.65	0.87**	0.75*
β-xanthin		0.76*	0.24	0.42	0.14	0.88**	−0.47	0.82**	0.77*
Betalain			0.29	0.48	0.12	0.88**	−0.49	0.88**	0.82*
T carotenoids				0.92**	0.95**	0.53	0.67*	0.74*	0.96**
β-carotene					0.98**	0.68*	0.72*	0.83**	0.92**
Vitamin C						0.32	0.35	0.82*	0.88*
TPC							0. 78*	0.98**	0.84**
TFC								0.87**	0.89**
TAC (DPPH)									0.97**

T carotenoids, Total carotenoids; TPC, Total polyphenol content (GAE µg g^−1^ dw); TFC, Total flavonoid content (RE µg g^−1^ dw); TAC (DPPH), Total antioxidant capacity (DPPH) (TEAC µg g^−1^ dw); TAC (ABTS^+^), Total antioxidant capacity (ABTS^+^) (TEAC µg g^−1^ dw); *significant at 5% level, **significant at 1% level, (n = 6).

In conclusion, at MSS and SSS conditions, leaf color parameters and pigments, vitamins, phenolic acids, flavonoids and antioxidant capacity of *A. tricolor* leaves were very high compared to control condition. Hence, salt-stressed *A. tricolor* leaves had a good source of natural antioxidants compared to plant grown in normal cultivation practices. The correlation coefficient revealed strong antioxidant activity of leaf pigments, β-carotene, vitamin C, TPC, TFC that could be contributed as a valuable food source for human diets and health benefit. *A. tricolor* cultivated under salinity stress could be contributed as a high quality product in terms of leaf pigments, bioactive compounds, vitamins, phenolic acids, flavonoids and antioxidants. It can be a promising alternative crop for farmers, especially in salt affected areas and also coastal belt in the world.

## Methods

### Experimental site, Plant materials and experimental conditions

Earlier, we collected 102 genotypes in different eco-geographical regions of Bangladesh. On the basis of our previous studies^14–21^, an antioxidant enriched high yield potential genotype (Accession VA13) was selected for this investigation. This genotype was grown in pots of a rain shelter open field of Bangabandhu Sheikh Mujibur Rahman Agricultural University, Bangladesh (AEZ-28, 24°23′ north latitude, 90°08′ east longitude, 8.4 m.s.l.). The seeds were sown in plastic pots (15 cm in height and 40 cm length and 30 cm width) in a randomized complete block design (RCBD) with three replications. N: P2O5:K2O were applied @92:48:60 kg ha^−1^ as a split dose. First, in pot soil, @46:48:60 kg ha^−1^ N: P2O5:K2O and second, at 7 days after sowing (DAS) @46:0:0 kg ha^−1^ N: P2O5:K2O. The genotype was grouped into three sets and subjected to four salinity stress treatments that are, 100 mM NaCl, 50 mM NaCl, 25 mM NaCl, and control or no saline water (NS). Pots were well irrigated with fresh water every day up to 10 days after sowing (DAS) of seeds for proper establishment and vigorous growth of seedlings. Imposition of salinity stress treatment was started at 11 DAS and continued up to 40 DAS (edible stage). Saline water (100 mM NaCl, 50 mM NaCl and 25 mM NaCl) and fresh water were applied to respective pots once a day. At 40 DAS the leaves of *Amaranthus tricolor* were harvested. All the parameters were measured in six samples.

### Chemicals

Solvent: methanol and acetone. Reagents: Standard compounds of pure phenolic acids, HPLC grade acetonitrile and acetic acid, vitamin C, gallic acid, rutin, methanol, DPPH (2,2-diphenyl-1-picrylhydrazyl), ABTS^+^ (2,2-azinobis-3-ethylenzothiazoline-6-sulphonic acid), trolox (6-hydroxy-2,5,7,8-tetramethyl-chroman-2-carboxylic acid), aluminum chloride hexahydrate, sodium carbonate, potassium acetate, Folin-Ciocalteu reagent, Caesium chloride, dithiothreitol (DTT) and potassium persulfate. All solvents and reagents used in this study were of high purity laboratory products obtained from Kanto Chemical Co. Inc. (Tokyo, Japan) and Merck (Germany).

### Leaf color measurement

The color parameters L*, a* and b* were measured by a color meter (TES-135A, Plus, Taiwan) with 15 replications. The value of L* indicates lightness, a* indicates the degree of red (+a*) or green (−a*) color, and b* indicates yellow (+b*) or blue (−b*) color. The C* value expressed as chroma indicates leaf color intensity calculated as Chroma C* = (a^2^ + b^2^)^1/2^.

### Determination of β-cyanin and β-xanthin content

β-cyanin and β-xanthin were extracted from fresh amaranth leaves using 80% methanol containing 50 mM ascorbic acid according to Sarker and Oba^32^. β-cyanin and β-xanthin were measured spectrophotometrically using a Hitachi U1800 instrument (Hitachi, Tokyo, Japan) at 540 and 475 nm, respectively. Results were expressed as the nanogram of betanin equivalent per gram of vegetable material fresh weight for β-cyanin and nanograms indicaxanthin equivalent per gram of *A. tricolor* fresh weight for β-xanthin.

### Determination of total carotenoids

Total carotenoids were determined from 80% acetone extracts of the fresh *A. tricolor* leaves following Sarker and Oba^33^ method spectrophotometrically using a Hitachi U1800 instrument (Hitachi, Tokyo, Japan) at 663, 646 and 470 nm for chlorophyll *a*, chlorophyll *b* and total carotenoids, respectively. Data were expressed as mg total carotenoids per 100 g fresh weight.

### β-carotene

The extraction and estimation of β-carotene were performed according to the protocol described by Sarker & Oba^32^. During the extraction process, 500 mg of fresh leaf sample was grounded in 10 ml of 80% acetone and centrifuged at 10,000 rpm for 3–4 min. The supernatant was taken in a test tube and the absorbance was measured at 510 nm and 480 nm spectrophotometrically using a Hitachi U1800 instrument (Hitachi, Tokyo, Japan). Data were expressed as mg β-carotene per g fresh weight.

The β-carotene content was calculated using the following formula:

$$\begin{array}{c}{\rm{Amount}}\,{\rm{of}}\,{\rm{\beta }} \mbox{-} \mathrm{carotene}=7.6({\rm{Abs}}{\rm{.}}\,{\rm{at}}\,480)-1.49({\rm{Abs}}{\rm{.}}\,{\rm{at}}\,510)\\ \,\,\,\,\,\,\,\,\,\,\,\times \,{\rm{Final}}\,{\rm{volume}}/(1000\times {\rm{fresh}}\,{\rm{weight}}\,{\rm{of}}\,{\rm{leaf}}\,{\rm{taken}})\end{array}$$

### Vitamin C

The total vitamin C defined as ascorbic acid (AsA) and dehydroascorbate (DHA). It was assessed by spectrophotometric detection on fresh plant tissues. The assay is based on the reduction of Fe_3_^+^ to Fe_2_^+^ by AsA and the spectrophotometric (Hitachi, U-1800, Tokyo, Japan) detection of Fe_2_^+^ complexes with 2, 2-dipyridyl^34^. DHA is reduced to AsA by pre-incubation of the sample with dithiothreitol (DTT). The absorbance of the solution was measured at 525 nm spectrophotometrically using a Hitachi U1800 instrument (Hitachi, Tokyo, Japan). Data were expressed as mg vitamin C per 100 g fresh weight.

### Extraction of samples for TPC, TFC and TAC analysis

Amaranth leaves were harvested at the edible stage (40 Days after sowing) which was air dried (In shade) for chemical analysis. One gram of dried leaves from each sample was grounded and suspended in 40 ml of 90% aqueous methanol in a tightly capped bottle (100 ml), which was then placed in a shaking water bath (Thomastant T-N22S, Thomas Kagaku Co. Ltd., Japan) for 1 h. The extract was filtered for further analytical assays of total polyphenol content, total antioxidant activity, total flavonoids content.

### Determination of total polyphenols (TPC

The total phenolic content of *A. tricolor* was determined using Folin-Ciocalteu reagent method described by Khanam *et al*.^26^ with gallic acid as a standard phenolic compound. 50 µl of the leaf extract solution was placed in a test tube along with 1 ml of Folin-Ciocalteu reagent (previously diluted 1:4, reagent: distilled water) and then mixed thoroughly. After 3 min, 1 ml of Na_2_CO_3_ (10%) was added, and the mixture allowed to stand for 1 h in the dark. The absorbance was measured at 760 nm spectrophotometrically using a Hitachi U1800 instrument (Hitachi, Tokyo, Japan). The concentration of total phenolic compounds in the leaf extracts was determined using an equation obtained from a standard gallic acid graph. The results are expressed as μg gallic acid equivalent (GAE) g^−1^ dw.

### Determination of total flavonoid content (TFC)

The total flavonoid content of *A. tricolor* extract was determined using aluminum chloride colorimetric method described by Khanam *et al*.^26^. For this assay, 500 µl of leaf extract was transferred to a test tube along with 1.5 ml of methanol, 0.1 ml of 10% aluminum chloride, 0.1 ml of 1 M potassium acetate and 2.8 ml of distilled water. After 30 min at room temperature, absorbance of the reaction mixture was measured at 415 nm spectrophotometrically using a Hitachi U1800 instrument (Hitachi, Tokyo, Japan). Rutin was used as the standard compound, and TFC is expressed as μg rutin equivalent (RE) g^−1^ dw.

### Total antioxidant capacity (TAC)

Antioxidant activity was measured using the diphenyl-picrylhydrazyl (DPPH) radical degradation method^26^. Briefly, 10 µl of leaf extract solution was placed in test tubes along with 4 ml of distilled water and 1 ml of 250 µM DPPH solution. The tubes were mixed and allowed to stand for 30 min in the dark before the absorbance was read at 517 nm spectrophotometrically using a Hitachi U1800 instrument (Hitachi, Tokyo, Japan). For the ABTS^+^ assay, method described by Khanam *et al*.^26^ was followed. The stock solutions included 7.4 mM ABTS^+^ solution and 2.6 mM potassium persulfate solution. The working solution was prepared by mixing the two stock solutions in equal quantities and allowing them to react for 12 h at room temperature in the dark. A 150 μl sample of leaf extract was allowed to react with 2850 μl of ABTS^+^ solution (1 ml ABTS^+^ solution mixed with 60 ml methanol) for 2 h in the dark. The absorbance was taken at 734 nm spectrophotometrically against methanol using a Hitachi U1800 instrument (Hitachi, Tokyo, Japan). Antioxidant activity was calculated as the percent of inhibition of DPPH and ABTS^+^ relative to the control using the following equation:

$${\rm{Antioxidant}}\,{\rm{activity}}\,( \% )=({\rm{Abs}}{\rm{.}}\,{\rm{blank}}\,-\,{\rm{Abs}}{\rm{.}}\,{\rm{sample}}/{\rm{Abs}}{\rm{.}}\,{\rm{blank}})\times 100$$

where, Abs. blank is the absorbance of the control reaction [10 µl methanol for TAC (DPPH), 150 μl methanol for TAC (ABTS^+^) instead of leaf extract] and Abs. sample is the absorbance of the test compound. Trolox was used as the reference standard, and the results were expressed as μg trolox equivalent g^−1^ dw.

### Extraction of samples for HPLC and LC-MS analysis

One gram of fresh-frozen leaves was homogenized with 10 ml of 80% methanol containing 1% acetic acid. The homogenized mixture was filtered through a 0.45 µm filter using a MILLEX^®^-HV syringe filter (Millipore Corporation, Bedford, MA, USA) and centrifuged at 10,000 g for 15 min. The final filtrate was used to analyze phenolic acids and flavonoids.

### HPLC analysis of phenolic acids and flavonoids

The amounts of phenolic acids and flavonoids in *A. tricolor* leaf sample were measured using HPLC with the method described by Khanam *et al*.^26^. The HPLC system (Shimadzu SCL10Avp, Kyoto, Japan) was equipped with LC-10Avp binary pumps, a degasser (DGU-14A) and a variable Shimadzu SPD-10Avp UV–vis detector. Phenolic acids and flavonoids were separated by a CTO-10AC (STR ODS-II, 150 × 4.6 mm I.D., Shinwa Chemical Industries, Ltd., Kyoto, Japan) column. The binary mobile phase consisted of 6% (v/v) acetic acid in water (solvent A) and acetonitrile (solvent B) was pumped at a flow rate of 1 ml/min for a total run time of 70 min. The system was run with a gradient program: 0–15% B for 45 min, 15–30% B for 15 min, 30–50% B for 5 min and 50–100% B for 5 min. The injection volume was 10 μl while the column temperature was maintained at 35 °C. The detector was set at 254, 280 and 360 nm for simultaneous monitoring of hydroxybenzoic acids, hydroxycinnamic acids and flavonoids. The compound was identified by comparing their retention time and UV–vis spectra with those of standards. The phenolic acids and flavonoids were also qualitatively confirmed using mass spectrometry. The sum of concentrations of all phenolic acids and flavonoids, quantified by HPLC, was denoted as the total phenolic index (TPI). From the HPLC data, TPI was obtained according to the method described by Khanam *et al*.^26^. All samples were prepared and analyzed in duplicate. The results were expressed as µg g^−1^ fresh weight (FW).

The Mass spectrometry analyses were performed in the negative ion mode using a JEOL AccuTOF (JMS-T100LP, JEOL Ltd., Tokyo, Japan) mass spectrometer fitted with an Agilent 1100 Series HPLC system and a UV–vis detector coupled on-line with an ElectroSpray Ionization (ESI) source. The column elutes were recorded in the range of m/z 0–1000. Needle voltage was kept at −2000 V. The chromatographic conditions were optimized to obtain chromatograms with good resolution of adjacent peaks, for which a slight modification was made in the method reported by Khanam *et al*.^26^. Extract constituents were identified by LC-MS-ESI analysis.

### Statistical Analysis

The data was statistically analyzed by analysis of variance (ANOVA) using Statistix 8 software and the means were compared by Duncan’s Multiple Range Test (DMRT) at 1% level of probability. The results were reported as the mean ± SD of three separate replications.

### Ethical Statement

The lab and field experiment in this study were carried out as per guidelines and recommendations of “Biosafety Guidelines of Bangladesh” published by Ministry of Environment and Forest, Government of the People’s Republic of Bangladeshi (2005).

## Data Availability

Data used in this manuscript will be available to the public.
